# The Jigger

**Published:** 1860-07

**Authors:** I. Rowell


### ARTICLE XXII.

*The Jigger.* Read before the San Francisco Medico-Chirurgical Society, by I. Rowell, M. D.

There is probably no insect that afflicts the human subject, about which so little is known by medical men generally, as the one named at the head of this article; and yet in those sections of country where they abound—viz. Central America, the West India Islands, and in Texas, and some other states of the American Union, they are a great terror and scourge to some classes of the inhabitants. The name is derived from the Spanish *chiquito* small, and is expressive of its diminutive size. This insect, which, when newly born, is invisible to the unaided eye, penetrates the human skin, where it feasts and fattens in safety, and grows to an enormous size, compared with its former proportions. Another and most dangerous quality of this insect is its faculty of propagation, by which it literally fills the unfortunate victim with a numerous family of young jiggers, causing the most intense irritation, inflammation, and swelling. I have seen the natives of Central America crippled for life; their lower extremities distorted with the most hideous deformity and alive with jiggers of all ages and sizes, from the freshly lain eggs of the insect to the full-grown jigger. Nothing but amputation affords any relief in this stage of this distressing malady. To what size they will grow, or what length of time they will live in the human flesh, I am unable to give even an approximate opinion.

Some seven years since, I removed one from the thigh of a laborer in the city of San Francisco, about six months subsequent to his time of service on the Panama railroad, where he was supposed to have contracted the insect. This was the largest that I ever saw, being at least one-fourth of an inch in thickness, and from an inch and a quarter to an inch and three-eighths in length, and tapering at each extremity like the finished end of a cigar. The part was swollen and inflamed, and appeared externally like an ordinary boil. It lay encysted just beneath and perpendicular to the surface. I carefully removed the cyst, which, according to New Grenadian surgery, contains the ovaria, which, if left behind, would soon be hatched into a numerous family of jiggers more dangerous than the parent stock. This patient soon left the city, and I never learned whether he was further troubled with jiggers.

The next case which came under my observation was Mr. A. B., drayman, a resident of San Francisco for the last seven years, aged 40, of good health. He had been engaged in cutting wood in the neighborhood of Lone Mountain Cemetery. He came to my office January 20th, 1860, complained of a burning pain on the surface in the epigastric region. On examination of the part it was found to be painful to pressure, with a bright areola extending some two inches around a central puncture like a bee-sting. I told him he had probably been bitten by some poisonous insect, ordered it painted with tinct. iodine sat., and gave the subject no further consideration. Twenty days subsequently, (Feb. 10th,) he came complaining of a similar pain on the back nearer the lower dorsal vertebræ. This presented the same appearance as the former without the puncture in the centre. On pressing, it gave to my finger a slight sense of fluctuation, and a burning lancinating pain to the patient. He told me that he had suffered this burning itching pain, with redness and swelling of the surface, since he first called on me; and that the swelling had moved once and a half round his body during the interim. A puncture through the skin in the centre and left portion of the swollen part, followed by pressure on either side of the opening, brought forth a live jigger one-eighth of an inch in thickness, and three-fourths of an inch in length. As Mr. B. has not been engaged in cutting fire-wood for twenty days past, he believes, and with reason, that the insect has been an inhabitant of his flesh ever since his first call, and that he has burrowed once and a half around his body. It was not, like the former, encysted. This is the first native California jigger that I have met with. In its habits it appears to differ from the Central American, being migratory. I report this case, hoping others may add their experience upon this apparently trifling but really important subject.

[There is very little known of the jigger by medical men generally who have never been in the region of country where they abound; but a more important subject could not occupy the attention of the medical profession of the present day. The immense amount of traveling through the tropical regions where persons are liable to contract this insect, and going thence to other remote regions of the globe might suffer beyond measure, or even die without the cause being suspected.]—*Editor San Francisco Medical Press.*

### Another Account by Joseph Haine, M. D.

At a meeting of the Medico-Chirurgical Association, the subject of the jigger was discussed, and several of the members expressed a wish to extend their knowledge of it. And the remarks made during the discussion led me to believe that altogether too little attention had been paid to it by medical journals, particularly of this coast, considering its great practical value to medical men and travelers to and from the Atlantic States through the tropical regions. In 1849, being about fifteen days from Callao, on board the Belgian ship *Charles Quint*, bound for San Francisco, one of the passengers complained of having felt at the bottom of his foot, near the big toe, a kind of itching, which had become somewhat painful. I examined the foot, and observed a dark blue round spot about three lines in diameter. There was no swelling nor redness around this spot, which had the appearance of some foreign body having entered the foot. On opening it, a drop of brown liquid came out, and after a little pressure a kind of reddish semi-transparent pouch as big as a sweet pea. This pouch bursted and little eggs similar to those of the fly came out in a great quantity. Some of the sailors being present at this little operation performed on deck, cried out, "Doctor, that is a jigger, put immediately cigar ashes into the wound, or the man will lose his leg." Instead of ashes I used nitrate of silver; in three days, the patient complaining of severe pain, I opened the little wound, and on pressure another quantity of eggs came out. I repeated the cauterization, two or three days after in pressing the wound, a few more eggs came out. I repeated the cauterization a third time, and healing took place. After this time several of the passengers in examining their feet, found out that they also were in possession of jigger's eggs, which were destroyed by the same process; the only difference being that two of them had them on the big toe right under the nail.

Returning from Europe in 1854 I crossed the Isthmus of Nicaragua. About two months after my arrival in San Francisco, I felt for a few days an itching sensation, not unpleasant, on my big toe, and happening by chance to look at it, discovered a black spot, similar to that of the jigger, but could hardly believe that after about three months, I would find the jigger's progeny in my own toe, there was no swelling, no redness, nothing but a black spot just under the beginning of the nail. I opened it with a lancet, and on pressing was not a little surprised to see the pouch coming out. I tried to extract it without bursting it, but in vain; plenty of eggs were visible, held together by a sort of mucuos liquid. By hard pressure I thought to have seen the last of them, and cauterized the wound, which gave me a burning sensation throughout the day. The next day my toe was somewhat swollen, and a slight suppuration had taken place; through pressure several more eggs came out. For three days I had the same sensation of burning, with redness and swelling of the toe. I cauterized every day; finally the wound healed. I counted over four hundred eggs.

A bright little daughter of the well known actor Ned Bingham, residing at the house of one of my patients, was brought to me to be examined; I was informed that she had returned with her father from the Nicaragua Walker expedition, where several of her family had died and her father been wounded. She complained of sore feet. I dont exactly remember how long it was after her return from Nicaragua. On examining her feet, I found black spots on the soles, which I had no doubt were jigger's nests, and one of them I was convinced of it. They all contained large quantities of eggs; I believe I opened as many as fifty, not only at the bottom of the feet, but on and between the toes, and several on her legs. I lost sight of her after a few days, she was taken away by her father. Since that time I have never had anything to do with jiggerdom. Till now I have never seen the insect called jigger, nor have I spoken with any person having seen it, nor can I give any description of it. A certain fact is that I have seen numbers of its eggs.—*San Francisco Medical Press.*

